# Hospital Problems in India

**Published:** 1920-10-02

**Authors:** 


### HOSPITAL PROBLEMS IN INDIA.

**Hospitals of the Frontier War.**

THE VICEROY has been in Wazirestan, and has visited the British General Hospital, the Fort Hospital, and No. 38 General Hospital. Lord Chelmsford expressed himself greatly impressed by the excellency of the arrangements, and cordially congratulated the medical and nursing staff.

**Child Welfare in India.**

IN connection with the successful Karachi Maternity and Child-Welfare Exhibition, which has been attracting large crowds during the mail week, an appeal has been issued for Rs. 75,000 to start two day nurseries, chiefly for the benefit of women workers in the coal, wool, hide, and skin trades

**Cholera in India.**

THE statement issued on mail day states that there were 881 deaths from cholera and 997 deaths from smallpox in all India for the previous week.

**Hospital for Children.**

AT the meeting of the Reception Committee in Bombay it was decided that the memorial to the Duke of Connaught's visit should be a hospital for children.

**Plague Mortality.**

THE plague mortality in India was particularly small during mail week. The Sanitary Commissioner with the Government of India states that the number of deaths last month approximately were 2,662. Although this is in excess of the total for 1919, it compares very favourably with the month for the last twenty-two years.

